# Acquisition of Drug Resistance and Dependence by Prions

**DOI:** 10.1371/journal.ppat.1003158

**Published:** 2013-02-07

**Authors:** Anja M. Oelschlegel, Charles Weissmann

**Affiliations:** 1 Department of Infectology, Scripps Florida, Jupiter, Florida, United States of America; 2 Department of Neuroscience, Scripps Florida, Jupiter, Florida, United States of America; University of Alberta, Canada

## Abstract

We have reported that properties of prion strains may change when propagated in different environments. For example, when swainsonine-sensitive 22L prions were propagated in PK1 cells in the presence of swainsonine, drug-resistant variants emerged. We proposed that prions constitute quasi- populations comprising a range of variants with different properties, from which the fittest are selected in a particular environment. Prion populations developed heterogeneity even after biological cloning, indicating that during propagation mutation-like processes occur at the conformational level. Because brain-derived 22L prions are naturally swainsonine resistant, it was not too surprising that prions which had become swa sensitive after propagation in cells could revert to drug resistance. Because RML prions, both after propagation in brain or in PK1 cells, are swainsonine sensitive, we investigated whether it was nonetheless possible to select swainsonine-resistant variants by propagation in the presence of the drug. Interestingly, this was not possible with the standard line of PK1 cells, but in certain PK1 sublines not only swainsonine-resistant, but even swainsonine-dependent populations (i.e. that propagated more rapidly in the presence of the drug) could be isolated. Once established, they could be passaged indefinitely in PK1 cells, even in the absence of the drug, without losing swainsonine dependence. The misfolded prion protein (PrP^Sc^) associated with a swainsonine-dependent variant was less rapidly cleared in PK1 cells than that associated with its drug-sensitive counterpart, indicating that likely structural differences of the misfolded PrP underlie the properties of the prions. In summary, propagation of prions in the presence of an inhibitory drug may not only cause the selection of drug-resistant prions but even of stable variants that propagate more efficiently in the presence of the drug. These adaptations are most likely due to conformational changes of the abnormal prion protein.

## Author Summary

Prions consist of PrP^Sc^, an aggregated conformer of the host protein PrP^C^. PrP^Sc^ multiplies by catalyzing the conformational conversion of PrP^C^ into a likeness of itself. Prions present as distinct strains that have the same primary amino acid sequence but differ in their conformation. Many distinct mouse-derived prions strains, for example RML, 22L or Me7, have been isolated. Prions can adapt to their environment. We investigated whether propagation of swainsonine-sensitive RML prions in the presence of the drug would yield swainsonine-resistant variants. Interestingly, this was not possible with a standard line of neuroblastoma cells, but in certain sublines not only swainsonine-resistant, but even swainsonine-dependent populations (i.e. that propagated more rapidly in the presence than in the absence of the drug) could be isolated. Once established, they could be propagated “indefinitely” in PK1 cells, even in the absence of the drug, without losing swainsonine dependence. In summary, our paper shows that propagation of prions in the presence of an inhibitory drug may not only cause the selection of drug-resistant prions but even of stable prion variants that propagate more efficiently in the presence of the drug. These adaptations are most likely due to conformational changes of the abnormal prion protein.

## Introduction

The prion, the transmissible agent mediating spongiform encephalopathies consists mainly if not entirely of PrP^Sc^, an aggregate of conformers of the host protein PrP^C^ (cellular prion protein). PrP^Sc^ may present in a proteinase K (PK)-sensitive and a PK-resistant form, designated PrP^sen^ and PrP^res^, respectively. Replication of PrP^Sc^ occurs by seeded aggregation (for reviews, see [1]–[4]).

Murine prions occur in form of many strains that can be distinguished by their cell tropism and susceptibility to drugs, as determined by the extended cell panel assay (ECPA) [5]–[9]. Prion populations exhibit features of Darwinian evolution in that they are subject to “mutations” that give rise to heterogeneity and allow selective amplification of prions in different environments. Brain-derived 22L prions are “R33 competent”, that is, they can infect R33 cells, and “swa resistant”, meaning that they can infect PK1 cells in the presence of the inhibitor swainsonine (swa), but after propagation in PK1 cells for several generations they become R33-incompetent and swa sensitive. However, when swa-sensitive 22L prions were propagated in PK1 cells in the presence of the drug, the prion population became swa resistant [6], [10]. Similarly, RML prions acquired quinacrine resistance in mice [11] and yeast prions resistance to epigallocatechin-3-gallate [12].

While brain-derived 22L prions are swa resistant, RML prions, both brain- or cell-derived, are swa sensitive [6], [7] (Figure S1), raising the question whether RML prions can nonetheless become swa resistant. We cultured RML-infected PK1 cells in the presence of swa under various conditions but repeatedly failed to obtain swa-resistant RML prions. Interestingly however, three PK1 subclones, AMO10, AMO18 or CAB19-2E4, when infected with RML and cultured in presence of swa, yielded prions that were fully swa-resistant or even “swa-dependent”, i.e. propagated more efficiently in the presence of swa than in its absence. AMO10-derived swa-resistant prions, when passaged through mouse brain, gave rise to a novel strain, distinct from the original RML, as assessed by the Extended Cell Panel Assay (ECPA).

These experiments suggest that in some RML-infected PK1-derived cell lines, but not in the PK1 cell line itself, the swa-sensitive prion populations contain a low level of variants, which can be selected in the presence of the drug to yield swa-resistant RML populations. We speculate that some cell line-dependent feature, be it the glycosylation state of PrP^C^ or the availability of some factor modulating its conformation, is responsible for the distinct behavior of some PK1 subclones in permitting the emergence of swa resistance or dependence.

## Materials and Methods

### Ethics statement

When working with mice all efforts were made to minimize suffering. This study was carried out in accordance with the recommendations in the Guide for the Care and Use of Laboratory Animals of the National Institute of Health. The protocol was approved by the Institutional Animal Care and Use Committee (The Scripps Research Institute – Scripps Florida Institutional Animal Care and Use Committee (TSRI-SF IACUC)).

### Cells

**Figure 1:**
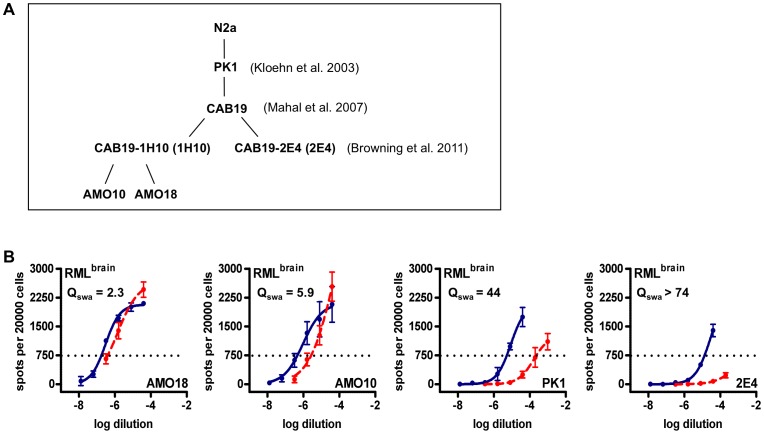
Different subclones of PK1 cells mediate susceptibility to swainsonine to different extents. **A.** Genealogy of PK1-derived subclones. **B.** Standard Scrapie Cell Assay of brain-derived RML prions on AMO18, AMO10, PK1 and 2E4 cells in the absence (blue line) or presence (red, dashed line) of swainsonine (swa; 1 µg/ml). RIs are the reciprocals of the dilutions required to yield 750 PrP^res^-positive cells per 20000 cells. Q_swa_, the ratio of RI_cell_ vs. RI_cell+swa_, indicates the inhibitory effect of swa on the propagation of RML prions in the individual cell lines.

The isolation of N2a-PK1 cells (PK1 for short) [13], CAD5 cells (CAD for short) [5], and R33_2H11_ cells [6] has been described. CAB19-1H10 cells (1H10 for short) and CAB19-2E4 cells (2E4 for short) were subclones of PK1-derived CAB19 cells [5], [14]. AMO10 and AMO18 cells were subclones of CAB19-1H10 cells (Figure 1A). All cell lines were propagated in OBGS (Opti-MEM [Invitrogen] containing 4.5% Bovine Growth Serum [Hyclone, Logan, UT], 50 units penicillin/ml and 50 µg streptomycin/ml [Invitrogen]). Uninfected cells were maintained for nine serial passages by 1∶10 splits before being replaced by freshly thawed cells.

### Prion strains

RML (RML 1856-II) was originally obtained from the Prion Unit, University College London and further propagated in CD1 and C57BL/6 mice (from Charles River Laboratories, Wilmington, MA).

### PIPLC

PIPLC was purified from E.coli transformed with a B.thuringiensis PI-PLC expression plasmid [15], [16]. The final preparation was not toxic to PK1 cells up to a level of at least 1 unit/ml.

### Prion propagation in mice

Mice were anesthetized by isoflurane inhalation and inoculated in the prefrontal cortex with 20- to 30-µl samples. For the preparation of stock brain homogenates, 30 µl of a 1% brain homogenate were injected. For the propagation of experimentally derived samples inocula were adjusted to deliver similar amounts of PrP^res^ (proteinase K-resistant PrP), as determined by western blot analysis. Clinical signs of disease included cessation of nesting, ruffled coat, lateral deviation with medial pronation of hind limbs, hind limb weakness, myoclonus, urinary incontinence with lesions in the vaginal area, hunched back, decreasing activity with increasing periods of lethargy, weight loss, squinty eyes, bruxing, shivering or trembling.

When clinical signs reached the terminal stage, the animals were euthanized by CO_2_ asphyxiation followed by cervical dislocation. The brains were collected and 10%-homogenates in PBS were prepared as described [6].

### Concentrated conditioned medium

Prions secreted by infected cells were concentrated from conditioned medium (CM). CM was cleared at 500× g for 5 minutes, centrifuged for two hours over a 10-ml sucrose cushion (20% sucrose [wt/wt] in 1× TNE buffer [25 mM Tris-HCl, pH 7.5; 150 mM NaCl; 1 mM EDTA]), in a Ti45 rotor (Beckman Coulter) at 35000 rpm and 4°C. The resulting pellet was suspended in OBGS to a 100 to 300-fold concentration of the original volume. It has been previously shown that the SSCA results obtained with prions from cell lysates or from CM are similar (Supporting Online Material in ref. [17]).

### Standard Scrapie Cell Assay (SSCA)

The procedure was as originally described [13], with some modifications: Six serial 1∶5 dilutions of the prion preparation (brain homogenate or concentrated conditioned medium) were added in triplicate or quadruplicate to 96-well plates and 5000 cells were added to each well. Triplicate or quadruplicate wells with uninfected cells served as background control. Another set of triplicates or quadruplicates contained the highest concentration of inoculum used as well as 10 µg pentosan polysulfate (Bene PharmaChem GmbH & Co. KG, Geretsried, Germany)/ml to inhibit prion replication [18] and to assess possible persistence of inoculum. After four days the cells were split 1∶5 to 1∶8, depending on their growth rate. After reaching confluence following the third split, 20000 cells/well were transferred into wells of pre-activated Multiscreen IP96-well 0.45-µm filter plates (Millipore). Supernatants were drained by vacuum, the plates were dried at 50°C for at least 1 hour and the samples were subjected to the PK-Elispot Assay either directly or after storing at 4°C.

### PK-Elispot Assay

Samples were incubated for 90 minutes at 37°C with 70 µl of 1 µg proteinase K (Roche)/ml lysis buffer (50 mM Tris-HCl, pH 8.0; 150 mM NaCl; 0.5% sodium deoxycholate; 0.5% TritonX-100). All further steps were carried out at room temperature. The samples were washed twice with PBS and denatured with 120 µl of 3 M guanidinium thiocyanate in 10 mM Tris-HCl, pH 8.0 for 10 minutes. After four washes with dH_2_O, samples were incubated for 1 hour with 0.5% non-fat dry milk in TBS (10 mM Tris-HCl, pH 8.0; 150 mM NaCl), followed by 1 hour of incubation with 70 µl of 0.7 µg humanized anti-PrP antibody D18 [19]/ml of 1% non-fat dry milk in TBST (10 mM Tris-HCl, pH 8.0; 150 mM NaCl; 0.1% Tween 20). After four washes with TBST, 70 µl of AP-conjugated anti-IgG (1∶5000, Southern Biotechnology Associates, Birmingham, AL) in 0.5% non-fat dry milk in TBST was applied for 1 hour. Wells were washed four times with TBST. Then the whole plate was immersed once in TBS and dried. Signals were visualized with AP Conjugate Substrate Kit (BioRad) and PrP^res^-positive cells (“spots”) were counted using the Bioreader 5000-Eb (BioSys).

### Extended Cell Panel Assay (ECPA)

Prion strains can be distinguished by their specific ability to infect a panel of different cell lines, as monitored by the SSCA [5]. Prion- and cell line-specific inhibitors like swainsonine, kifunensine or castanospermine [7] as well as curcumine [20], compound B [21] or Congo Red [20], [22] complement and extend the scope of the original panel of cell lines. In the work described here prion characteristics were analyzed using CAD cells, R33_2H11_ cells and PK1 cells, as well as PK1 cells in the constant presence of 1 µg swainsonine (Logan Natural Products)/ml, 5 or 10 µg kifunensine (Cayman Chemicals)/ml, 0.25 µg curcumine (Sigma-Aldrich)/ml, 1.6 ng compound B (a gift from K. Doh-ura, Tohoku University Graduate School of Medicine, Japan)/ml or 108.4 ng CongoRed (Sigma-Aldrich)/ml. The cells' response to prions is characterized by the Response Index (RI), the reciprocal of the dilution required to yield a designated number of positive cells (“spots”) per 20000 cells; the ratio of RIs is characteristic for different prion strains. We characterize swa sensitivity, swa resistance or swa dependence of the different prion samples by the ratio Q_swa_ = RI_PK1_/RI_(PK1+swa)_. Because Q_swa_ (Q for short) may vary from one assay to another, Q of a sample was compared relative to that of RML, determined in the same assay, to obtain Q_rel_ = Q_sample_/Q_RML_ and prions were defined as being swa sensitive if Q_rel_≥1; as semi-resistant if 1>Q_rel_>(1/Q_RML_); as resistant if Q_rel_ = (1/Q_RML_) and as “swa dependent” if Q_rel_<(1/Q_RML_).

### Frequency Assay

CM recovered from brain-derived RML-infected PK1 or AMO10 cells, was used to infect PK1 cells in the presence (undiluted CM) or absence (five serial 1∶2 dilutions, starting from undiluted CM) of swa. Pools of 2000 cells (in the presence of swa) or of 10 cells (plus 1990 uninfected cells, in the absence of swa) were distributed into the wells of 96-well plates, grown to confluence and propagated for six splits. The plates were then assayed by the SSCA and wells containing PrP^res^-positive cells (spot numbers>[background+5 SDs]) were scored as “putative positive”. We have found that PrP^res^-positive cells may persist in the presence of swa even though the prions are not swa resistant (because inhibition by swa is not complete). Therefore seven each of AMO10-derived and PK1-derived “putative positive” prion populations were analyzed by the SSCA on PK1 cells in the absence or presence of swa to establish “true” swa resistance. From the percentage of validated positive samples, the average number of prions per well (m_w_) could be determined as m_w_ = ln(1/P_mw_(0)), where P_mw_(0) = 1-(positive wells/total wells) and N = cells/well, and hence the average number of prions per cell m_c_ = m_w_/N. The ratio m_c(+swa)_, prions/cells in the swa-containing plate, to m_c(no swa)_, prions per cell in the swa-free plate, yields the frequency of pre-existing swa-resistant prions in the population.

### Half-life experiments

In order to compare the degradation rate of different brain- or cell-derived prion samples PK1 cells were seeded in 6-well plates at 5.7×10^4^ cells in 1.4 ml media and inoculated with CM (final concentration: 30 fold) from infected cells, or with 10% RML brain homogenate (final dilution: 2×10^−4^). After four days, cells were split; 0.9×10^6^ cells were transferred to a 15-cm culture dish, grown for four days and split again by transferring 1.4×10^6^ cells each into two 15-cm dishes, to one of which 2 µg swa/ml were added. Three days after drug application, cells were split a third time; 10^6^ cells each were transferred to ten 15-cm dishes, resulting in 10 dishes with and 10 without swa. On the following day, PIPLC was added to a final concentration of 1 unit/ml to four of 10 dishes. At time zero, before adding PIPLC, two dishes were harvested, and thereafter one dish per condition after 7, 12, 18 and 24 hours. Cells, recovered in culture media, were centrifuged at 3000× g and 4°C for 5 min. The pellet was re-suspended in 0.5 to 1 ml of PBS, the cells were counted, centrifuged again at 3000× g and 4°C for 5 min and finally frozen in −80°C as suspension of 2.5×10^7^ cells/ml in PBS. Lysates were prepared by three freeze-thaw cycles using liquid nitrogen, followed by several passages through a 28-gauge syringe. All samples were adjusted to 1.6 mg protein/ml in PBST (PBS, 0.5% TritonX-100) and digested with 20 µg proteinase K (Roche)/ml at 37°C for 1 hour. After terminating digestion with 2 mM PMSF, the samples were denatured by heating in XT-MES sample buffer (BioRad) at 100°C for 10 minutes and were subjected to electrophoresis on triplicate gels with subsequent western blot analysis as described below. After correcting for differences in luminescence intensity between the gels, the intensity of PrP^res^ at each time point was normalized to the PrP^res^ intensity at the zero-hour time point and plotted as a function of time on a log scale.

### Conformational Stability Assay

Samples (30 µg total protein/ml) were adjusted to guanidine hydrochloride (Gdn.HCl) in 10 mM Tris-HCl (pH 8.0) in a dilution series ranging from 0.2 M to 4.2 M. After 15 minutes at 25°C the Gdn.HCl concentration was brought to 0.2 M in all samples and volumes were equalized. This was followed by digestion with 0.6 µg proteinase K (Roche)/ml 0.5% TritonX-100 in PBS (PBST) for 1 hour at 37°C. The digestion was stopped with 2 mM PMSF. Proteins were precipitated by TCA (10% final concentration), incubated 30 minutes on ice and centrifuged at 16000× g for 15 min at 4°C. The pellets were resuspended in cold acetone, centrifuged again and finally resuspended in PBST. After addition of XT-MES sample buffer (BioRad) samples were denatured at 100°C for 10 minutes and subjected to electrophoretic separation on triplicate gels with subsequent western blot analysis as described below.

### Western blot analysis

To detect PrP^res^ by western blot analysis, samples were, if not stated otherwise, subjected to standard PK digestion: 3 mg total protein/ml in PBST were digested with 25 µg proteinase K (Roche)/ml at 37°C for 1 hour. Undigested controls were run in parallel. The digestion was stopped with 20 µl 100 mM PMSF/ml and the samples were denatured by boiling in XT-MES sample buffer (BioRad) at 100°C for 10 minutes. Electrophoretic separation (4–12% Criterion gel, BioRad) and wet transfer (BioRad) to PVDF Immobilon membranes (Millipore) were performed by standard procedures. All further steps were performed at room temperature. Membranes were incubated for 1 hour in blocking solution (5% non-fat dry milk in PBST) and immunostained with 0.7 µg humanized anti-PrP antibody D18/ml in 1% non-fat dry milk in PBST (PBS; 0.1% Tween 20), followed by three washes with PBST and 1 hour of incubation with HRP-conjugated anti-IgG antibody (Southern Biotechnology Associates, Birmingham, AL, 1∶15000 in 0.5% non-fat dry milk in PBST). After three washes with PBST, chemiluminescence was induced by ECL-Plus (Pierce) and recorded by CCD imaging (BioSpectrum AC Imaging System; UVP).

## Results

Prion strains can be distinguished by their ability to infect a panel of different cell lines [5], as monitored by the Standard Scrapie Cell Assay (SSCA) [13]. The response of a cell line to a prion strain preparation is measured by the Response Index (RI), the reciprocal of the dilution required to yield a specified proportion of positive cells. Prion strains are characterized by distinct RI ratios on different cell lines and by susceptibility to inhibitors [5], [8], [9].

Swainsonine (swa), an inhibitor of α-mannosidase II, causes misglycosylation of N-glycosylated proteins, including PrP^C^, which carries up to two N-linked glycans. Infection of neuroblastoma N2a-derived PK1 cells with RML in the presence of swa reduces the RI by 1.5 to 2 logs but has no inhibitory effect on other cell lines tested [7].

We quantify susceptibility of a prion strain to swa by determining the RI on PK1 cells in the absence or presence of swa and calculating the quotient Q = RI_PK1_/RI_(PK1+swa)_. Because Q may fluctuate from one assay to another, we determine Q of a sample relative to that of RML, determined in the same assay, to obtain Q_rel_ = Q_sample_/Q_RML_. We define prions as being swa sensitive if Q_rel_≥1; semi-resistant if 1>Q_rel_>(1/Q_RML_); resistant if Q_rel_ = (1/Q_RML_) and “swa dependent” if Q_rel_<(1/Q_RML_).

### Search for swa-resistant RML prions

In several preliminary experiments PK1 cells were infected with RML and split repeatedly in the presence of swa, or PK1 cells were repeatedly exposed to concentrated conditioned medium (CCM) of RML-infected PK1 cells containing secreted prions [10]. Although in some instances RML-exposed PK1 cells remained infected when propagated in the presence of the drug, the lysate or CCM of the cells was unable to infect PK1 cells in the presence of the drug, as judged by the SSCA (Figure S2A). We concluded that the apparent swa resistance came about not because swa-resistant prions had emerged but because swa, although decreasing RML prion propagation in PK1 cells by about 1.5–2 logs [7], did not completely abrogate it and therefore allowed propagation of infected cells. Subclones of PK1 cells may vary considerably in their properties, such as susceptibility to infection by RML or 22L prions [5] or their ability to mediate swa inhibition [7].

**Figure 2:**
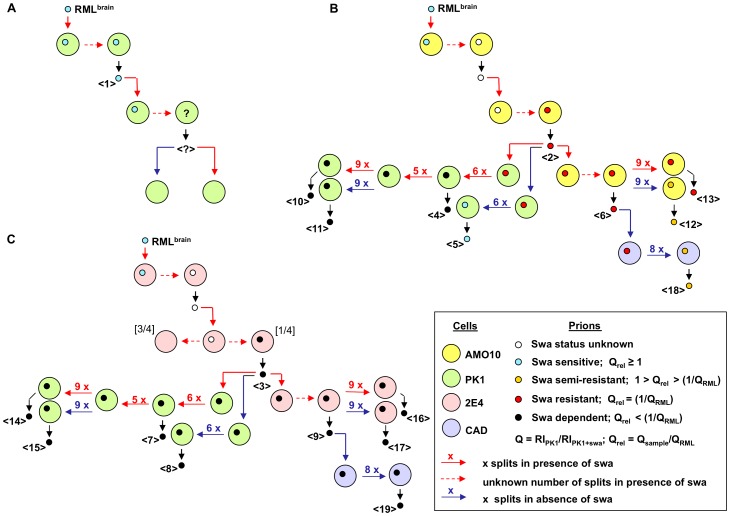
Scheme displaying the emergence of swa-resistant and swa-dependent RML prions, and their transmission through various cell lines. Initially, PK1 or PK1-derived cells were infected with RML prions, cultured in the presence of swa, and prions secreted into the conditioned medium (CM) were concentrated (CCM) and used to infect fresh batches of cells, also in the constant presence of swa. This cycle was repeated at least once more. The resulting prions were further propagated under various conditions. Large circles indicate cell lines; small circles indicate prions; horizontal arrows represent propagation of infected cells and vertical arrows transfer of prions; <#> indicates a prion sample whose swa resistance is reported in Table 1 and/or in one of the further figures. **A.** CCM from PK1 cells (large green disks), collected after the first infection (<1>) in the presence of swa (red arrows), contained low titers of swa-sensitive prions (small blue disks). After transfer to a fresh batch of PK1 cells in the presence of swa, infectivity dropped below detectability (<?>). **B.** AMO10 cells (large yellow disks) infected with RML in the presence of swa yielded swa-resistant prions (small red disks) (<2>); upon further transmission to AMO10 cells (large yellow disks) in the presence of swa the prions remained swa resistant; upon transmission to PK1 cells (large green disks) in the presence of swa the prions developed swa dependence. When transferred in the absence of swa the prions became swa sensitive (small, blue disks), semi-resistant (small orange disks) or swa dependent (small black disks) depending on whether they had previously been cultured with (red arrows) or without swa (blue arrows), and on the cell line (CAD cells violet disks). **C.** In the case of 2E4 cells, swa-dependent prions (small black disks) were recovered after the first transfer of CCM in the presence of swa (<3>); these prions remained swa dependent regardless of culture conditions or cell line.

**Figure 3:**
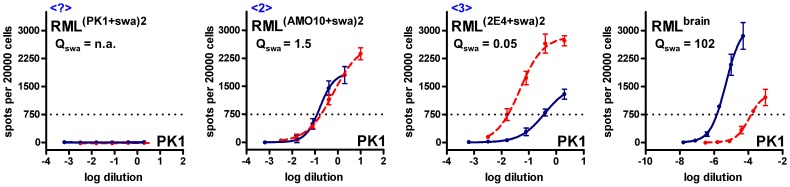
Swa-resistant and swa-dependent RML prions can only be selected in certain cell sublines. PK1 cells and cells of the PK1-derived sublines AMO10 and 2E4 were infected with RML prions, cultured in the presence of swa for 2 splits, and prions secreted into the conditioned medium (CM) were concentrated (CCM) and used to infect fresh batches of cells in the constant presence of swa (see scheme in Figure 2). CCM recovered from these cultures was analyzed by the SSCA on PK1 cells in the absence (blue line) or presence (red, dashed line) of swa. The proportion of PrP^res^ positive cells was plotted against the logarithm of the inocula dilutions and RIs were determined as reciprocal of the dilutions required to yield 750 PrP^res^ positive cells per 20000 cells. Q_swa_, the ratio of RI_cell_ vs. RI_cell+swa_, indicates the inhibitory effect of swa on the analyzed prion sample, to be compared with swa-sensitive, brain-derived RML prions (rightmost graph). Prions could not be detected in CM recovered from PK1 cells (<?>). Prions recovered from AMO10 cells (<2>) were found to be swa resistant, that is, they were propagated to the same extent in the presence or absence of swa. Prions recovered from 2E4 cells (<3>) were “swa dependent”, i.e. were propagated more efficiently in the presence of swa. n.a., not applicable.

We considered that PK1-derived cell lines (Figure 1A) which mediate swa susceptibility to a lesser degree than PK1, such as AMO10 and AMO18 might be more likely to allow selection of swa-resistant mutants than CAB19-2E4 (2E4 for short), which showed a more pronounced swa effect (Figure 1B). As shown schematically in Figure 2, we infected PK1 (A), AMO10 (B) and 2E4 cells (C) with RML in the presence of swa, and after the cells had reached confluence the conditioned medium (CM) was used to infect fresh cells in the presence of swa. The prions in the CM recovered after this second round of infection were assayed for swa resistance by the SSCA on PK1 cells. As shown in Figure 3, no infectivity was recovered from PK1 cells (Table 1, <?>), whilst prions propagated in AMO10 cells in the presence of swa were virtually fully swa resistant (Figure 3, Table 1, <2>). Unexpectedly, prions propagated in 2E4 cells in the presence of swa (Figure 3, Table 1, <3>), were not only swa resistant, but “swa dependent”, that is, they scored even higher in the presence of swa than in its absence, giving a quotient Q = RI_PK1_/RI_PK1+swa_ of 0.05, as compared to 102 for RML^brain^ prions that served as controls.

**Table 1:** Relative swa resistance of various prion populations.

Prion population^a^				
Origin	Number^b^	Figure	Designation	Q_rel_ c×10^3^
brain derived			RML^brain^	1000
cell derived	<?>	3	RML^(PK1+swa)2^	nid
	<1>		RML^(PK1+swa)1^	nrd
	<2>	3	RML^(AMO10+swa)2^	15
	<3>	3	RML^(2E4+swa)2^	0.49
	<4>	5	RML^(AMO10+swa)2/(PK1+swa)6x^	4.6
	<5>	5	RML^(AMO10+swa)2/(PK1)6x^	>220
	<6>	7	RML^(AMO10+swa)3^	19
	<7>	5	RML^(2E4+swa)2/(PK1+swa)6x^	1.1
	<8>	5	RML^(2E4+swa)2/(PK1)6x^	0.86
	<9>	7	RML^(2E4+swa)3^	0.41
	<10>	5	RML^(AMO10+swa)2/(PK1+swa)20x^	0.33*
	<11>	5	RML^(AMO10+swa)2/(PK1+swa)11x→(PK1)9x^	0.25*
	<12>	4	RML^(AMO10+swa)2/(AMO10+swa)→(AMO10)9x^	5.5*
	<13>	4	RML^(AMO10+swa)3^	1.8*
	<14>	5	RML^(2E4+swa)2/(PK1+swa)20x^	0.19*
	<15>	5	RML^(2E4+swa)2/(PK1+swa)11x→(PK1)9x^	0.091*
	<16>	4	RML^(2E4+swa)3^	0.21*
	<17>	4	RML^(2E4+swa)2/(2E4+swa)→(2E4)9x^	0.22*
	<18>	6	RML^(AMO10+swa)3/(CAD)8x^	110
	<19>	6	RML^(2E4+swa)3/(CAD)8x^	0.24
brain derived	<AMO10-brain-a>	8	RML^(AMO10+swa)3/brain1^	460
	<AMO10-brain-b>	8	RML^(AMO10+swa)3/brain2^	670
	<2E4-brain-a>	8	RML^(2E4+swa)3/brain1^	670
	<2E4-brain-b>	8	RML^(2E4+swa)3/brain2^	810
	<2E4-brain-c>	8	RML^(2E4+swa)3/brain3^	810

a Prion samples were assayed by the Standard Scrapie Cell Assay (SSCA) on PK1 cells in the absence or presence of swainsonine (swa, 1 µg/ml).

b as shown in Figure 2 (cell-derived samples) or Figure 8 (brain-derived samples).

c Q_rel_ = Q_sample_/Q_RML_ where Q = RI_PK1_/RI_PK1+swa_ and RI_cell_ = reciprocal of the dilution required to yield specified designated number of PrP^res^ positive cells per 20000 cells.

nid = no infectivity detected; nrd = no ratio determinable.

**Figure 4:**
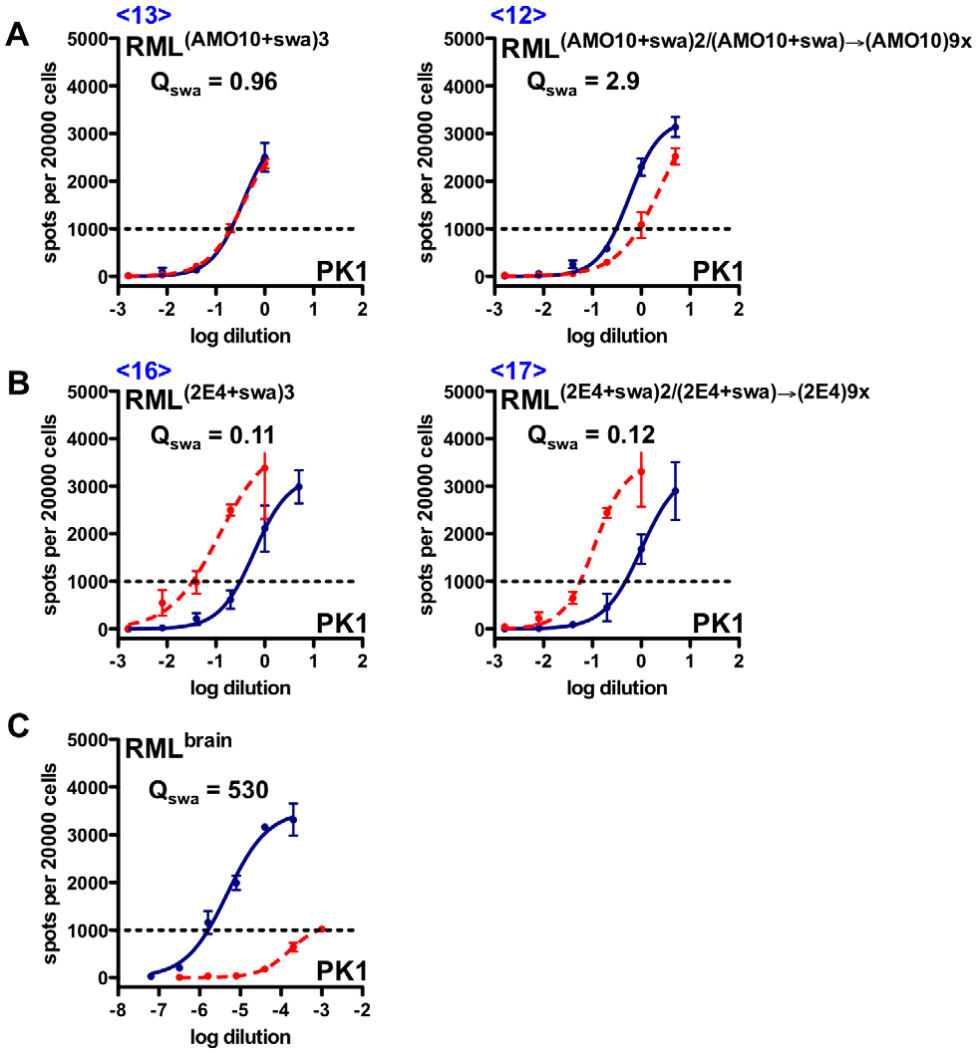
Standard Scrapie Cell Assay of swa-resistant and swa-dependent RML prions propagated in the presence or absence of swa. AMO10 and 2E4 cells were infected with RML prions, cultured in the presence of swa, and prions secreted into the conditioned medium (CM) were concentrated (CCM) and used to infect fresh batches of cells, also in the constant presence of swa. This cycle was repeated once more; the infected cells were cultured in the presence of swa for an extended period of time and then divided into two batches, of which one continued to be propagated in the presence of swa for nine splits while the other was propagated in parallel in the absence of swa (see Figure 2B, C). CCM from these cultures was then analyzed by the SSCA on PK1 cells in the absence (blue line) or presence (red, dashed line) of swa. RIs are the reciprocals of the dilutions required to yield 1000 PrP^res^ positive cells per 20000 cells. Q_swa_ = RI_cell_/RI_cell+swa_ indicates the inhibitory effect of swa. **A.** Swa-resistant AMO10-derived prions that were constantly propagated in the presence of swa (<13>) remained swa resistant, but reverted to semi-resistance when propagated for nine splits in the absence of swa (<12>). **B.** Swa-dependent 2E4-derived prions remained swa dependent whether propagated for 9 splits in the presence of swa (<16>) or in its absence (<17>). **C.** Swa-sensitive, brain-derived RML prions. Q_swa_ for RML^brain^ was unusually high in this assay and that of Figure 5B, leading to the low Q_rel_ values shown in Table 1.

**Figure 5:**
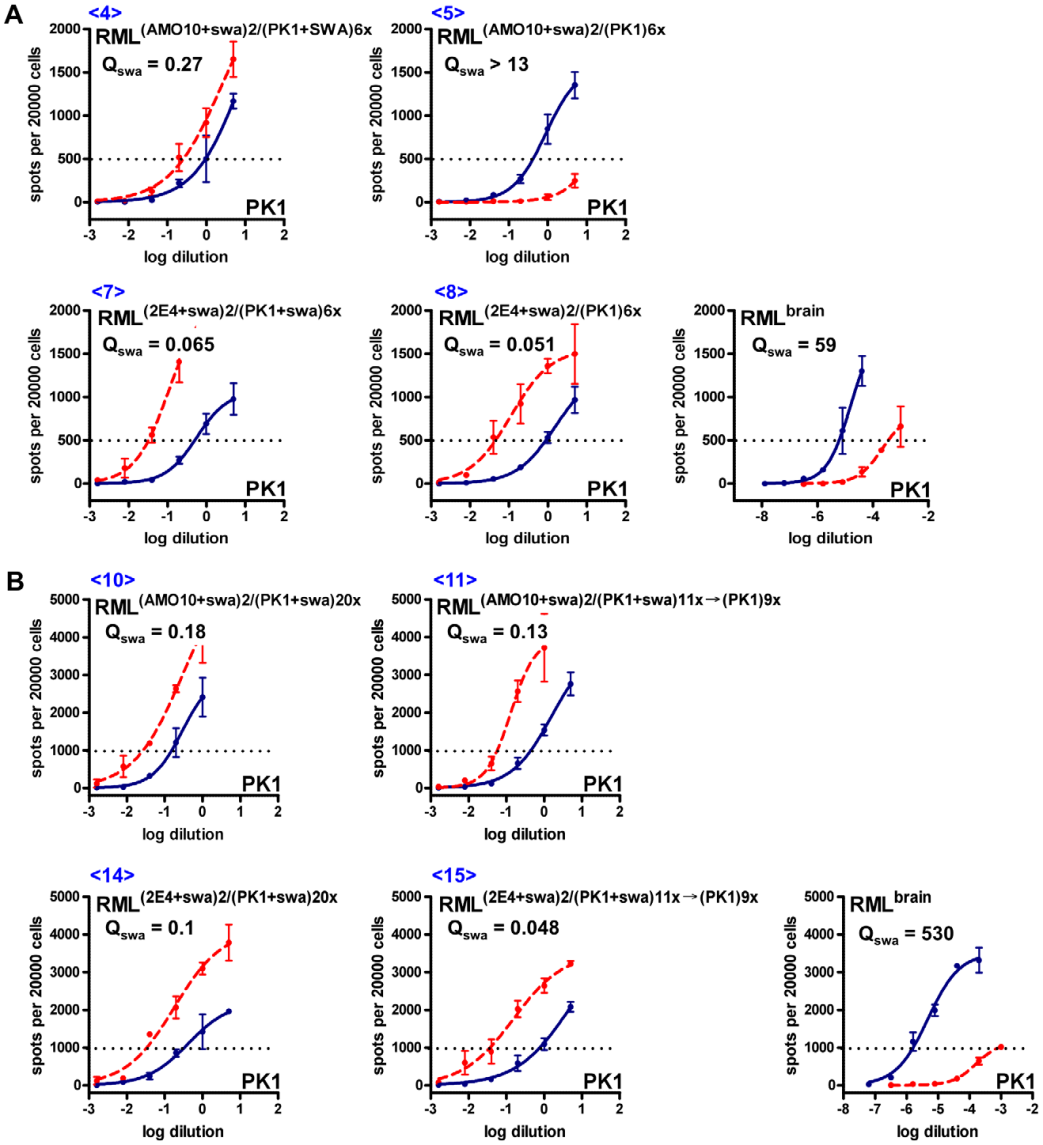
Standard Scrapie Cell Assay of swa-resistant and swa-dependent RML prions transferred to PK1 cells and propagated in the presence or absence of swa. AMO10 and 2E4 cells were infected with RML prions, cultured in the presence of swa, and prions secreted into the conditioned medium (CM) were concentrated (CCM) and used to infect fresh batches of cells in the constant presence of swa. This cycle was repeated once more, whereupon the prions were transferred to PK1 cells in the presence or absence of swa (Figure 2B, C). CCM was analyzed by the SSCA on PK1 cells in the absence (blue line) or presence (red, dashed line) of swa. **A.** Swa-resistant AMO10-derived prions developed swa dependence when propagated in PK1 cells in the presence of swa (<4>); when propagated in PK1 cells in the absence of swa, they reverted to swa sensitivity (<5>). In contrast, swa-dependent 2E4-derived prions remained swa dependent in PK1 cells, whether they were propagated in the presence (<7>) or absence (<8>) of swa. RIs are the reciprocals of the dilutions yielding 500 PrP^res^ positive cells per 20000 cells. Q_swa_ = RI_cell_/RI_cell+swa_ reflects inhibition by swa and may be compared to the value for swa-sensitive, brain-derived RML prions. **B.** AMO10- or 2E4-derived prions that had acquired or retained swa dependence after propagation in PK1 cells in the presence of swa were either continuously propagated in the presence of swa (<10>, <14>) or cultured for eleven splits in the absence of swa (<11>, <15>). Once swa dependent, they remained dependent, whether propagated in the presence or absence of swa. RIs are the reciprocals of the dilutions required to yield 1000 PrP^res^ positive cells per 20000 cells. Q_swa_ for RML^brain^ is unusually high in this assay and that of Figure 4C, leading to the low Q_rel_ values shown in Table 1.

* Q_RML_ was exceptionally high in the experiments of Figures 4C and 5B (about 2.7 rather than 1.5–2 logs), resulting in what may be misleadingly low values for Q_rel_ in the associated samples.

Prions propagated in AMO10 cells in the presence of swa remained swa resistant (Figure 4A, left, Table 1 <13>), but became semi-resistant when propagated for 9 splits in the absence of the drug (Figure 4A, right, Table 1 <12>). 2E4 cells continued to propagate swa-dependent prions even after nine splits in the absence of swa (Figure 4B, right, Table 1 <17>). In a repetition of the selection experiment, 3 rounds of selection in swa were required to obtain fully swa-resistant RML prions from AMO10 cells, at which point PK1 cells were again cured of infection. However, in this experiment only partially swa-resistant RML prions were obtained from 2E4 cells. Nonetheless, as shown below, the original isolate of swa-dependent RML prions (RML^(2E4+swa)^) could be propagated indefinitely in various cell lines.

RML-infected AMO18 cells, as was the case for AMO10 cells, also yielded swa-resistant RML prions after 2 rounds of propagation in swa. (Figure S3).

### Are swa resistance and swa dependence stable properties of the selected RML prions, and are they maintained after transfer to other cell lines?

In order to determine whether swa resistance and swa dependence were properties of the prions or of the cells that harbored them, we transferred the prions to PK1 cells and determined their properties. Swa-resistant RML prions from AMO10 cells, transferred to PK1 cells and passaged for 6 splits in the presence of swa, surprisingly gave rise to swa-dependent prions, whilst passaging in the absence of swa led to production of swa-sensitive prions (Figure 5A, top; Table 1, <4>, <5>). However, if the PK1 cells infected with swa-resistant RML prions were first passaged for 11 splits in the presence of swa, and then for 9 further splits, in either the presence or absence of swa, swa-dependent prions were produced (Figure 5B, top; Table 1, <10>, <11>).

Thus, swa-resistant prions in AMO10 cells were stably maintained in the presence of swa, but in its absence reverted to partial swa resistance or swa sensitivity. However, if transferred to PK1 cells and propagated in the presence of swa, the prions became swa dependent and no longer reverted to swa sensitivity when the drug was withdrawn. Interestingly, although PK1 cells failed to mediate the generation of swa-resistant prions, they were not only able to propagate them but allowed them to evolve to drug dependence.

Figures 4B and 5, and Table 1 show that 2E4 or PK1 cells infected with swa-dependent prions from 2E4 cells (Figure 2C, Table 1, <3>) continued to propagate swa-dependent prions with a Q = RI_PK1_/RI_PK1+swa_ quotient of 0.05–0.1, whether grown in the presence of swa (<7>, <14>, <16>) or in its absence (<8>, <15>, <17>).

**Figure 6:**
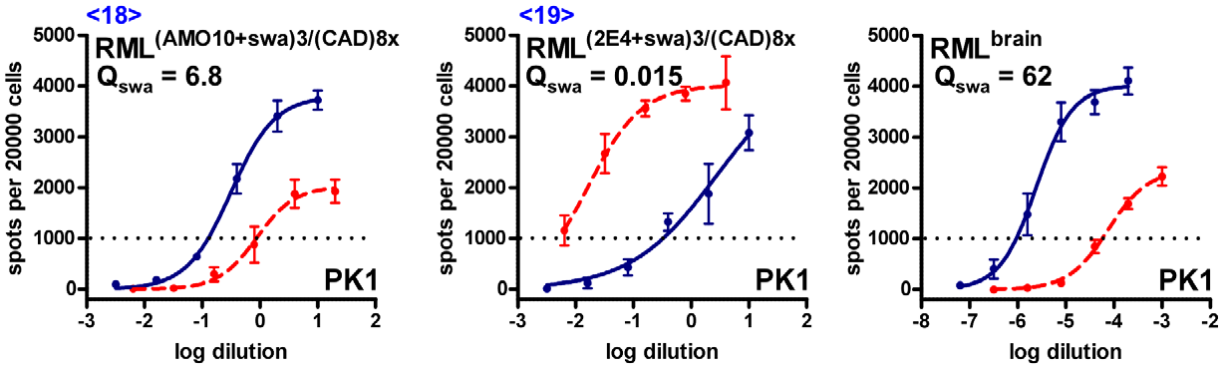
Standard Scrapie Cell Assay of swa-resistant and swa-dependent RML prions transferred to CAD cells in the absence of swa. Swa-resistant AMO10-derived prions and swa-dependent 2E4-derived prions were transferred to CAD cells and propagated in the absence of swa for 8 splits. Concentrated conditioned medium from these cultures was then analyzed by the SSCA on PK1 cells in the absence (blue line) or presence (red, dashed line) of swa. RIs are the reciprocals of the dilutions yielding 1000 PrP^res^-positive cells per 20000 cells. Q_swa_ = RI_cell_/RI_cell+swa_ reflects inhibition by swa and may be compared with the value for swa-sensitive, brain-derived RML prions (rightmost panel). Swa-resistant AMO10-derived prions reverted to swa sensitivity (<18>), while swa-dependent 2E4-derived prions remained dependent (<19>).

When transferred to and propagated in CAD cells in the absence of swa, 2E4-derived swa-dependent prions also retained their swa dependence (Figure 6, Table 1, <19>), whilst AMO10-derived swa-resistant prions became semi-resistant (Figure 6, Table 1, <18>).

In summary, when RML-infected PK1 cells were propagated in the presence of swa, the cells were cured of infection, whilst propagation of RML-infected AMO10 and 2E4 cells in the presence of swa led to the emergence of swa-resistant prions, or even of prions that replicated more efficiently in the presence of swa.

### Frequency Assay for swa-resistant RML prions

Previous work has supported the proposal that prion strain populations are quasi-species, consisting of a major conformer and multiple variants that are constantly selected against, but are replenished by mutation [1]. Within the framework of this hypothesis, and considering that AMO10 but not PK1 cells could give rise to swa-resistant prions, we hypothesized that RML-infected PK1 cells have a more restricted prion repertoire than AMO10 cells. In an attempt to substantiate this hypothesis we subjected prions from RML-infected PK1 and AMO10 cells (both infected and cultured in the absence of swa) to the frequency assay [1], [10], [17]: PK1 cells were infected in the presence or absence of swa with prions from the two sources, and pools of 2000 cells (in the presence of swa) or of 10 cells (plus 1990 uninfected cells, in the absence of swa) were distributed into the wells of 96-well plates, grown to confluence and propagated for six splits. The ratio of prion-infected cells in the swa-containing plate to that in the swa-free plate yields the frequency of pre-existing swa-resistant prions in the population. As shown in Table S1B, 8.7×10^5^ PK1 cells infected with RML-infected AMO10 cell supernatant in the presence of swa, assayed in pools of 2000 cells/well, yielded about 0.003% validated infected cells, while in the absence of swa (assayed in pools of 10 cells/well) an average of 4.1% infected cells were identified. Thus, in the AMO10 populations, about 0.08% of the prions were swa resistant prior to exposure to the drug. No cells containing swa-resistant prions were found among 8.8×10^5^ cells in the cognate experiment with the PK1 population, setting an approximate upper limit of 0.02%. This experiment showed that swa-resistant prions pre-existed in the AMO10-derived population but, because of the low numbers, it did not allow a statistically significant conclusion to be drawn regarding the PK1-derived population.

As an incidental note, while validating a set of 7 apparently positive clones from the AMO10-derived prions grown in PK1 cells in the presence of swa, we found three clones to be swa sensitive, two swa resistant and two swa dependent (Figure S2B), confirming the previous findings shown in Figures 2B and 5, namely that swa-resistant AMO10-derived prions can become swa dependent when passaged in PK1 cells in the presence of swa.

**Figure 7:**
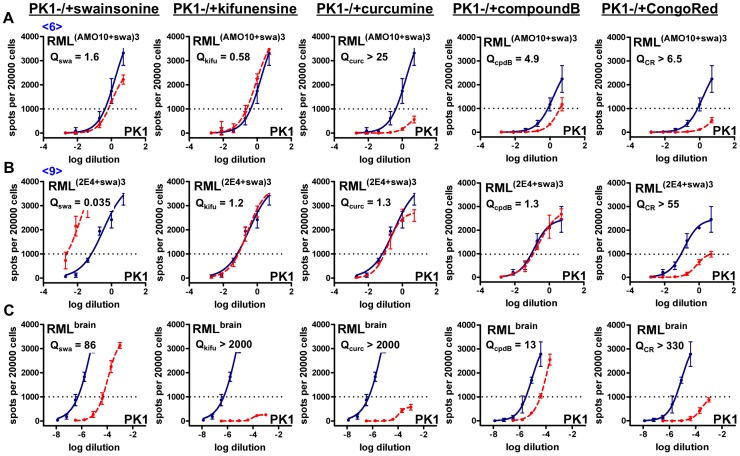
Standard Scrapie Cell Assay of swa-resistant and swa-dependent RML prions in the presence of various prion inhibitors. Swa-resistant AMO10-derived prions (<6>), swa-dependent 2E4-derived prions (<9>) and swa-sensitive, brain-derived prions were analyzed by the SSCA on PK1 cells in the absence (blue line) or presence (red, dashed line) of inhibitor. RIs are the reciprocals of the dilutions yielding 1000 PrP^res^-positive cells per 20000 cells. Q_drug_ = RI/RI_+drug_ indicates the inhibitory effect of a drug on the RI of a particular prion isolate (in PK1 cells) and may be compared with the Q_drug_ value of brain-derived RML prions. **A.** While swa-resistant AMO10-derived prions are also resistant to kifunensine (kifu), they are semi-resistant to compound B (cpdB) and sensitive to curcumine (curc) or Congo Red (CR). **B.** Swa-dependent, 2E4-derived prions are resistant to kifu, curc and cpdB, but inhibited by CR. **C.** The propagation of swa-sensitive brain-derived RML prions, used as control, is strongly inhibited by kifu, curc and CR.

As indicated in Figure 2B and C, AMO10-derived swa-resistant prions propagated in AMO10 cells in the presence of swa remained swa resistant for >10 splits (Table 1, <13>), and when passaged through PK1 cells in the presence of swa became swa dependent (Table 1, <10>), while 2E4-derived prions were swa dependent whether propagated in 2E4 or PK1 cells (Table 1, <16> and <14>). Although both AMO10- and 2E4-derived prions passaged in PK1 cells were R33 incompetent and resistant to kifunensine, they differed in that 2E4-derived prions were far more resistant to curcumine and distinctly more resistant to compound B than their AMO10-derived counterpart, as shown by the Extended Cell Panel Assay (ECPA; Table 2 and Figure 7).

**Table 2:** Extended Cell Panel Assay (ECPA) of swa-resistant and swa-dependent RML prions.

Inocula^a^	logRI @ 750 spots					logQ^b^ = log[RI_PK1_/RI_PK1+drug_]		Q^b^		Q_rel_ c×1000	
	CAD	2H11	PK1	PK1+swa	PK1+kifu	swa	kifu	swa	kifu	swa	kifu
**RML^(AMO10+swa)2/(PK1+swa)20x^**	1.4	<−1	0.79	2.7	1.0	−1.9	−0.24	0.013	0.58	0.81	<1.4
**RML^(2E4+swa)2/(PK1+swa)20x^**	1.3	<−1	0.75	2.5	0.91	−1.7	−0.16	0.019	0.69	1.1	<1.7
**RML^(AMO10+swa)3^**	1.8	<−1	0.27	0.51	0.80	−0.24	−0.53	0.58	0.30	35	<0.74
**RML^(2E4+swa)3^**	0.54	<−1	0.16	1.6	0.29	−1.5	−0.13	0.033	0.74	2.0	<1.9
**RML^brain^**	7.1	<3	5.6	4.4	<3	1.2	>2.60	17	>400	1000	1000

a Prion samples were assayed by the Standard Scrapie Cell Assay on CAD, R33_2H11_ (2H11) and PK1 cells, the latter in the absence or presence of swainsonine (swa; 1 µg/ml) or kifunensine (kifu; 5 µg/ml). The number of PrP^res^ positive cells per 20000 cells was plotted against the logarithm of the inocula dilutions and RIs were determined as reciprocals of the dilutions required to yield 750 PrP^res^ positive cells per 20000 cells.

b Q, the ratio of RIs (or logQ, the log of the ratios) in the absence and presence of a drug characterizes the drug sensitivity of a prion strain.

c Q_rel_ = Q_sample_/Q_RML_ where Q = RI_PK1_/RI_PK1+drug_.

**Table 3:** Rate of PrP^res^ depletion, synthesis and clearance in the presence or absence of swa in PK1 cells infected with swa-sensitive or swa-dependent RML prions.

Sample^a^	swa	PIPLC	(k_de_±SD)*10^3^	(k_s_±SD)*10^3^	(k_cl_±SD)*10^3^	(k_p_±SD)*10^3^
**PK1+ RML^brain^**	−	−	−0.54±2.7	79±8.5	−36±7.3	−44±3.4
	+	−	−21±1.9	65±10	−48±8.9	−38±3.5
	−	+	−80±5.9	0	−36±7.3	−45±4.2
	+	+	−84±8.3	0	−48±8.9	−36±3.3
**PK1+ RML^(AMO10+swa)/(PK1+swa)14x^**	−	−	−1.1±3.9	52±8.3	−12±7.1	−41±2.2
	+	−	−15±1.1	29±5.9	−8.7±4.8	−35±3.3
	−	+	−6 3±5.1	_0_	−12±7.1	−51±4.8
	+	+	−47±4.4	0	−8.7±4.8	−38±1.9

a PK1 cells were infected with brain-derived, swa-sensitive RML prions or swa-dependent prions (RML^(AMO10+swa)/(PK1+swa)14^, derived from RML propagated sequentially in AMO10 cells and PK1 cells in the presence of swa; similar to <10> in Figure 2B, however passaged in PK1 +swa for 14 rather than 20 splits). Cells were propagated for 8 days and seeded into two 15-cm dishes, to one of which 2 µg swa/ml were added. Three days after swa addition, cells were transferred to ten 15-cm dishes, resulting in 10 dishes with and 10 without swa. One day after the third split, two dishes were harvested (time zero) and 1 unit PIPLC/ml media was added to four dishes of each condition, following which one dish per condition was harvested after 7,12,18 and 24 hours. For each sample, cells were counted and PrP^res^ levels were determined by western blot analysis (see Figure 9). **k_de_** = PrP^res^ depletion rate; **k_s_** and **k_cl_** are the rates of PrP^res^ synthesis and clearance, respectively; **k_p_** is the rate of cell partitioning = negative of cell growth rate, and includes data points from 0 to 24 hours. **k_de_**, **k_s_** and **k_cl_** are calculated from the 7- to 24-hour points by the equation **k_de_** = **k_s_**+**k_cl_**+**k_p_**, with **k_s_** = 0 in the presence of PIPLC under the reasonable assumption that **k_cl_** is the same in the presence or absence of PIPLC. The experiment was evaluated from triplicate western blots. The Table shows that in PK1 cells the clearance rate of swa-dependent PrP^res^ was 3× lower in absence of swa, and 5.5× lower in the presence of swa than that of swa-sensitive RML (t-test evaluation:

* p = 0.015 and

** p = 0.0025, respectively).

### Propagation of swa-resistant and -dependent RML prions through mouse brain

Earlier experiments had shown that the changes incurred by brain-derived 22L prions after propagation in cell lines, namely transition to swa sensitivity and R33 incompetence, were abrogated after passaging in brain, and that the resulting prions were not distinguishable from the original 22L by the CPA [10], [17]. We therefore considered brain-adapted and cell-adapted 22L prions as substrains of 22L, because of their facile interconvertibility.

**Figure 8:**
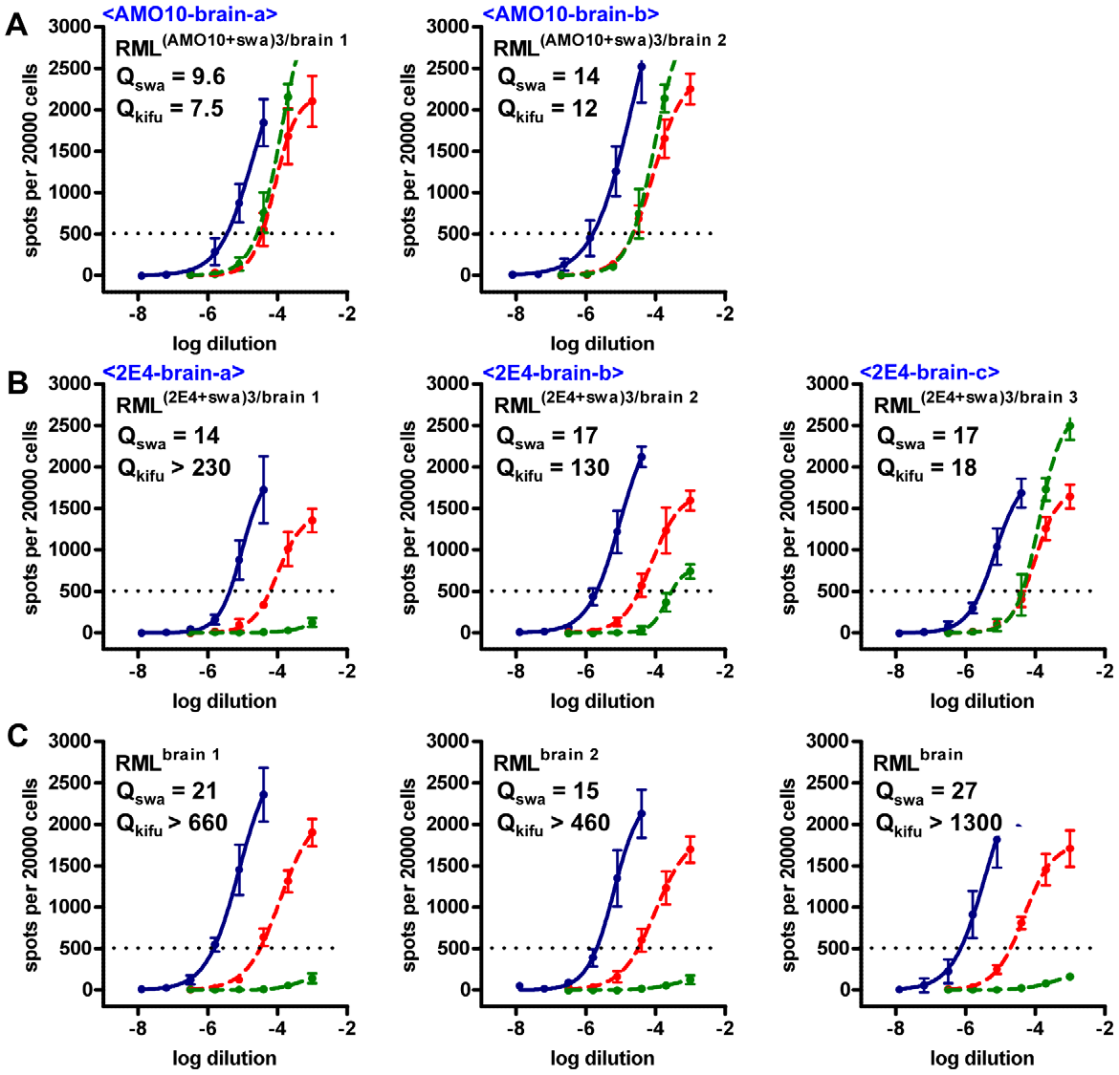
Standard Scrapie Cell Assay in the presence of various prion inhibitors (ECPA) of AMO10- and 2E4-derived RML prions after propagation in mice. Swa-resistant AMO10-derived prions, swa-dependent 2E4-derived prions and authentic brain-derived RML prions were propagated in C57BL6 mice and analyzed by ECPA on PK1 cells in the absence (blue line) or presence of either swa (red, dashed line) or kifu (green, dashed line). RIs are the reciprocals of the dilutions required to yield 500 PrP^res^ positive cells per 20000 cells. Q_drug_ = RI/RI_+drug_ indicates the inhibitory effect of the drug on the RI of a prion sample and may be compared with the Q_drug_ value of brain-derived RML prions. **A.** Brain-passaged AMO10-derived prions (<AMO10-brain-a>, <AMO10-brain-b>) were sensitive to swa, but semi-resistant to kifu. **B.** Brain-passaged 2E4-derived prions were inhibited by swa, however, one sample (<2E4-brain-c>) contained prions that were semi-resistant to kifu, one sample (<2E4-brain-b>) contained kifu-sensitive prions, and one sample (<2E4-brain-a>) contained prions that were strongly inhibited by kifu. **C.** Two experimental control brains (brain1 and brain2) of mice that were inoculated with authentic RML prions and a homogenate of pooled brains (lab stock) were analyzed as controls; the prions were swa-sensitive and strongly inhibited by kifu.

The situation with swa-resistant RML prions proved to be different. We inoculated swa-resistant AMO10-derived, swa-dependent 2E4-derived as well as authentic brain-derived RML prions into C57BL/6 mice. Although the resulting histopathological and immunohistochemical changes were indistinguishable, the ECPA (Figure 8 and Table 1) revealed that while brain-passaged AMO10-derived prions (<AMO10-brain-a>, <AMO10-brain-b>) had indeed reverted back to swa sensitivity (Q = 9.6 and 14, for duplicate brains), becoming similar to authentic RML prions (Q = 21, 15 and 27, for triplicate brains), they were largely resistant to kifu (Q = 7.5 and 12), in contrast to authentic RML prions, which were strongly inhibited by the drug (Q>661, >457, >1349, for triplicate brains). These results were confirmed by multiple independent assays. Thus, selection for swa resistance in cell culture followed by propagation in brain resulted in a mutant strain characterized by resistance to kifu but not to swa. Three brains inoculated with 2E4-derived prions gave different, albeit repeatable results in the ECPA: While swa was equally inhibitory to all three samples (Q = 14, 17, 17), one brain (<2E4-brain-c>) contained prions that were semi-resistant to kifu (Q = 18), one sample (<2E4-brain-b>) contained kifu-sensitive prions (Q = 130), albeit less sensitive than brain-derived RML prions (Q>661, >457, >1349, for triplicate brains), and the third brain (<2E4-brain-a>) contained prions that were as strongly inhibited by kifu as brain-derived RML (Q>230). Inasmuch as the mice were genotypically identical, the diverse development of the prion populations may be due to an incidental phenotypic characteristic of the mouse (such as its immunological status); another possibility is that the number of prions following injection was greatly reduced, leading to a “bottleneck” from which distinct populations emerged.

To determine whether the PrP^res^ associated with RML, AMO10- and 2E4-derived prions had distinct physico-chemical properties, we subjected the cognate infected brain homogenates to the conformational stability assay. As shown in Figure S4, the Gnd.HCl_1/2_, i.e. the guanidinium molarity at which 50% of the PrP^res^ became susceptible to PK digestion, was 1.4 M for all three preparations.

### Distinct clearance rates of PrP^res^ associated with wild-type and swa-dependent prions in PK1 cells

The level of PrP^res^ in a cell is determined by the rates of its synthesis and depletion. Depletion comes about by partitioning, and by clearance due to degradation and secretion. We determined the rate of depletion (k_de_) by arresting PrP^res^ synthesis with PIPLC [23], [24] and measuring cell number and PrP^res^ levels as a function of time. k_de_ = k_s_−k_p_−k_cl_ (1), where k_de_ is the rate of PrP^res^ decrease, k_s_ the rate of synthesis, k_cl_ the rate of clearance and k_p_ is the rate of cell division, whence for k_s_ = 0: k_de_ = −k_p_−k_cl_ (2). Under the assumption that k_p_ and k_cl_ are not affected by PIPLC treatment, we can estimate k_s_ from equation (1) under steady state conditions, when PrP^res^/cell is constant: k_de_ = k_s_−k_p_−k_cl_ = 0, from which k_syn_ = k_p_+k_cl_.

**Figure 9:**
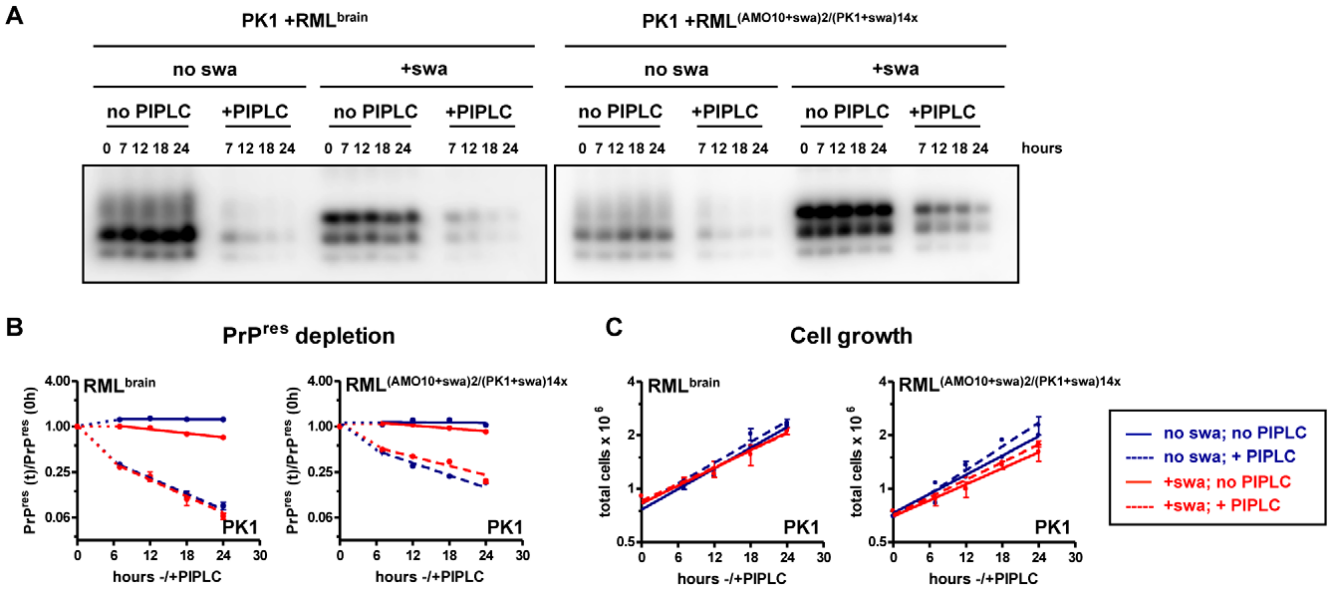
Depletion of PrP^res^ following inhibition of PrP^res^ synthesis in PK1 cells infected with either RML^brain^ or RML^(AMO10+swa)2/(PK1+swa)14x^. PK1 cells were infected with brain-derived, swa-sensitive RML prions or swa-dependent prions derived from RML propagated sequentially in AMO10 cells and PK1 cells in the presence of swa (RML^(AMO10+swa)/(PK1+swa)14x^), grown for 8 days and seeded into two 15-cm dishes to one of which 2 µg swa/ml were added. Three days after swa addition, the cells from each plate were transferred to ten 15-cm dishes, resulting in ten dishes with and ten without swa. One day after the third split, two dishes were harvested (time zero), 1 unit PIPLC/ml media was added to four dishes of each condition, and one dish per condition was harvested after 7, 12,18 and 24 hours. Cells were counted at each time point and equal amounts of protein were subjected to western blot analysis to determine PrP^res^ levels. From PrP^res^ level changes and growth rate PrP^res^ synthesis and PrP^res^ clearance were calculated as shown in Table 3. **A.** Western-blots showing PrP^res^ signals at indicated time points. Samples were loaded in triplicate gels; one representative blot is shown. **B.** After correction for differences in luminescence intensity between gels, the intensity of PrP^res^ at each time point was normalized to the PrP^res^ intensity at the 0 hour time point and plotted, beginning at the 7 hour time point, as a function of time on a log2 scale. **C.** In order to determine growth rate and partitioning rate (see Table 3), total cell numbers from each time point were plotted as a function of time on a log2 scale.

Figure 9 shows the PrP^res^ levels of PK1 cells infected with RML^brain^ and swa-dependent RML^(AMO10+swa)2/(PK1+swa)14x^ (similar to <10> in Figure 2B, however passaged in PK1 +swa for 14 rather than 20 splits), pre-treated or not for 4 days with swa, as a function of time after addition or not of PIPLC. For both prion samples the PrP^res^ levels remained constant in the absence of PIPLC. The rate of depletion, as measured after addition of PIPLC, appeared to be biphasic, with a rapid drop in the first 7 hours and a slower exponential decrease thereafter. After the initial 7-hour period, the clearance rate of PrP^res^ of swa-dependent RML in absence of swa was 3× lower than that of swa-sensitive RML, and was 5.5× lower in the presence of the drug, indicating that susceptibility of PrP^res^ to clearance was a major determinant for swa resistance.

## Discussion

It was not too surprising that swa-resistant 22L prions could be selected from a swa-sensitive, cell-adapted 22L population [10], because “natural” brain-derived 22L prions are swa resistant to begin with. It was however unpredictable that RML prions, which are swa sensitive, both when propagated in brain or in PK1 cells, would give rise to swa-resistant and even swa-dependent populations when passaged in the presence of swa.

What is the biochemical basis for swa sensitivity, resistance and dependence? Swa, by inhibiting α-mannosidase II, causes misglycosylation of N-glycosylated proteins, including PrP^C^ and PrP^Sc^ (PrP^res^). General misglycosylation could alter the cellular mechanisms involved in PrP^Sc^ synthesis and clearance; however it is more likely that most of the effect is related to the misglycosylation of PrP^Sc^ because different variants of RML prions exhibit a vast difference in their response to swa in the same cell line. How could misglycosylation of PrP^Sc^ lead to these effects? X-ray crystallographic studies on IgG1-Fc have demonstrated that the nature of the N-linked glycans may affect the conformation of a glycoprotein and thereby change its susceptibility to degradation [25]. Indeed, we found that swa-sensitive and swa-dependent RML PrP^res^ have different degradation rates. Moreover, misglycosylation may affect the interaction of PrP^Sc^ with other cellular proteins, such as lectin chaperones, and thereby modulate the rate of synthesis and/or clearance. If, as we propose, prions form quasi-species populations, a conformer present at low levels that is able to overcome a deleterious effect due to swa-mediated misglycosylation could be selected and give rise to swa-resistant or even -dependent PrP^res^ species.

A further point of interest is that swa and kifu resistance of RML prions do not necessarily go hand in hand: Although both drugs lead to misglycosylation, the resulting glycans are of different nature, namely hybrid-type glycans in the case of swa [26]–[28] and oligomannose-type glycans in the case of kifu [29]. This indicates that different types of misglycosylation might modulate the interactions of PrP^C^ and/or PrP^res^ with proteins involved in PrP^res^ synthesis or degradation in distinct ways. Castanospermine, an inhibitor of α-glucosidase 1, which removes glucose residues from the initial core and generates a high-mannose glycan, shows an inhibition pattern different from both swa and kifu [7].

**Figure 10:**
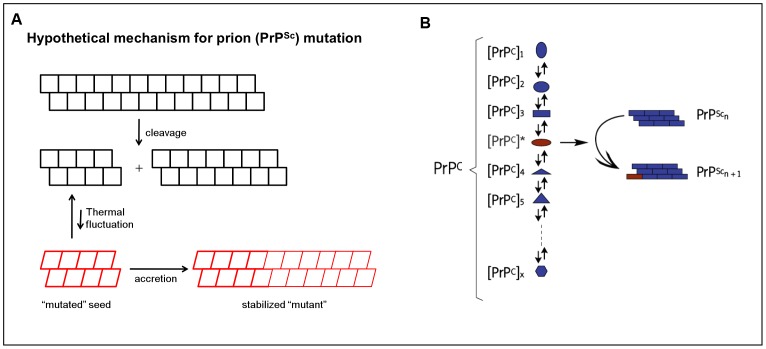
Hypothetical mechanisms for prion mutation and cell tropism. **A.** The conformation of a PrP^Sc^ unit in an aggregate may be “locked” even if it is not the thermodynamically most favored one. The shorter the aggregate, the more the conformation of its subunits may be subject to thermal fluctuation; accretion of PrP^C^ stabilizes the variant conformation, resulting in “mutation” of the prion. **B.** PrP^C^ may assume a variety of conformations, of which only one or a few can readily accrete to a particular seed. Different conformers may be present at different steady state concentrations and could be favored by the nature of the N-glycans and/or by interaction with cellular factors (such as small RNAs). Cell tropism may reflect the preponderance of different sets of conformations in distinct cell types. Panel A is from ref. [17] and Panel B from ref. [30].

Why was it possible to reproducibly generate swa-resistant prions in AMO10 and AMO18 cells (and at least once in 2E4 cells), but not in PK1 cells, although the latter were able to propagate swa-resistant prions once they were generated in other cell lines and even mediate their conversion to swa dependence? Within the framework of the quasi-species hypothesis, it is possible that the diversity of the prion population in PK1 cells is distinct from, or more restricted than that in AMO10 cells, as suggested by the frequency analysis for swa-resistant prions from RML-infected PK1 and AMO10 cells, so that there are no swa-resistant variants to be selected. This in turn begs the question as to why the diversity is different. We suggest that prions undergo mutations that are based on small, thermally induced conformational changes of short PrP^res^ aggregates [1], which are stabilized if and when appropriate (“competent”) PrP^C^ conformers accrete to it (Figure 10). Thus, as we proposed earlier to explain cell tropism [30], the repertoire of PrP^res^ variants could depend on the repertoire of PrP^C^ conformers, which in turn may be determined by the N-linked glycans, of which there is a great variety [31]–[33] or by association with some cell-derived molecule, for instance a small RNA. The affinity of PrP^C^ for RNA (reviewed in [34]) and the requirement for RNA in PMCA-mediated prion propagation in vitro [35]–[37] has been documented. Thus, if prion propagation at low concentrations of both swa-resistant seed and competent monomer follows 2^nd^ order kinetics, the rate of swa-resistant prion formation in PK1 cells may be lower than the replication rate of the cells, thereby preventing emergence of swa-resistant prions. On the other hand, elevated concentrations of swa-resistant seed in the same cells could promote rates of swa-resistant prion formation high enough to allow persistence in dividing cell populations and evolution of prions with increased swa resistance and even “dependence”.

Several prion strain variants we have described earlier, which resulted from the transfer of brain-derived 22L prions to different cultured cell lines, reverted to the original strain when returned to brain [10], [17], suggesting that the activation energy barriers between the various conformations were low [1]. In another instance, brain-derived 139A prions, when passaged through PK1 cells and returned to brain, became indistinguishable from the 79A/RML strain, suggesting that the activation energy barrier between the two strains was high in brain but low in PK1 cells, and that the 79A/RML conformers were preferentially replicated in the cells [8]. In the experiments described in this paper, at least one distinct swa-resistant and three swa-dependent RML variants were characterized, derived from AMO10 and from 2E4 cells, which, after passaging in PK1 cells, retained their properties even after propagation in the absence of swa. Upon propagation in brain, the swa-resistant (and kifu-resistant) prions from AMO10 cells reproducibly became swa sensitive, however were still semi-resistant to kifu, showing that a strain distinct from RML had emerged. Whether or not these prions would further change or revert to RML upon continued propagation in mouse brain was not determined. The 2E4-derived prions, when propagated in mouse brain, also became swa sensitive, however the resistance to kifu varied in the three brains tested, ranging from semi-resistance to full susceptibility; this may reflect very slow reversion to the original RML strain, which proceeded at slightly different rates in these brains.

In summary, our experiments have shown that RML prions, when subjected to selective pressure in cultured cells, can develop a variety of novel properties, which are stable in cell culture but are further modified when propagated in brain. The repertoire of possible conformations associated with PrP conformers with the same primary sequence appears to be unexpectedly vast.

## Supporting Information

Figure S1 **Brain and cell-derived RML prions are swa sensitive and R33 incompetent.** When assayed by ECPA on R33_2H11_ cells (green line) or on PK1 cells in the absence (blue line) or presence (red, dashed line) of swa, PK1 cell-derived and brain-derived RML prions show the same cell tropism pattern: Neither of the two prion populations is able to infect R33_2H11_ cells and their propagation in PK1 cells is similarly inhibited by the presence of swa. RIs are the reciprocals of the dilutions required to yield 750 PrP^res^ positive cells per 20000 cells. Q_swa_ = RI_PK1_/RI_PK1+swa_ indicates the inhibitory effect of swa on the analyzed prion sample.
(TIF)

Figure S2 **Validation of apparently swa-resistant prion populations recovered in the Frequency Assay on PK1- or AMO10-derived prions.** Cells from seven positive wells obtained in the Frequency Assay (Table S1A) on PK1-derived and AMO10-derived populations were expanded, and concentrated CM was analyzed by the SSCA on PK1 cells in the absence (blue line) or presence (red, dashed line) of swa. RIs are the reciprocals of the dilutions required to yield 750 PrP^res^ positive cells per 20000 cells. Q_swa_ = RI_PK1_/RI_PK1+swa_ reflects the inhibitory effect of swa on the analyzed prion sample and may be compared to the effect on brain-derived RML prions. **A.** All seven PK1-derived prion samples infected PK1 cells in the absence but not in the presence of swa. **B.** Three of the seven AMO10-derived prion populations were swa-sensitive prions (samples 8, 13, 14), two were swa resistant (samples 9, 10) and two were swa dependent (samples 11, 12). **C.** Brain derived RML prions were assayed as control.
(TIF)

Figure S3 **Selection of swa-resistant RML prions in PK18 cells.** AMO18 cells were infected with RML prions, cultured in the presence of swa, and prions secreted into the conditioned medium (CM) were concentrated (CCM) and used to infect fresh batches of cells in the constant presence of swa. CCM recovered from this culture was analyzed by the SSCA on R33_2H11_ cells (green line) as well as PK1 cells in the absence (blue line) or presence (red, dashed line) of swa (left graph). RIs are the reciprocals of the dilution yielding 1000 PrP^res^ positive cells per 20000 cells. Q_swa_ = RI_PK1_/RI_PK1+swa_ indicates the inhibitory effect of swa on the analyzed prion sample and may be compared to the Q_swa_ value of swa-sensitive brain-derived RML prions, assayed in parallel (right panel). Brain-derived RML prions are unable to infect R33_2H11_ cells and their propagation in PK1 cells is strongly inhibited by swa. PK18-derived prions are inefficiently propagated by R33_2H11_ cells but fully swa resistant.
(TIF)

Figure S4 **Conformational stability assay of brain homogenates.** Swa-resistant AMO10-derived prions and swa-dependent 2E4-derived prions as well as brain-derived RML prions were propagated in mice. Brain homogenates of the three samples were adjusted to increasing concentrations of guanidine hydrochloride (Gdn.HCl) ranging from 0.2 M to 4.2 M, incubated for 15 minutes at 25°C, treated with proteinase K and precipitated with trichloroacetic acid. PrP^res^ was detected by western blot analysis on triplicate gels, of which one representative blot is shown, and signals were expressed in percentage of the signal for 0.2 M Gnd.HCL. Gnd.HCl_1/2_, i.e. the molarity at which 50% of the PrP^res^ became susceptible to PK digestion, was 1.4 M for all three preparations.
(TIF)

Table S1 **Quantification of preexisting swa-resistant prions in PK1- and AMO10-derived prion populations by the Frequency Assay.** **A.** Conditioned medium recovered from PK1 and AMO10 cells, both inoculated with brain-derived RML prions in the absence of swa, was subjected to the Frequency Assay on PK1 cells: PK1 cells were infected in the presence or absence of swa with prions from one or the other source, and pools of 2000 cells (in the presence of swa) or of 10 cells (plus 1990 uninfected cells, in the absence of swa) were distributed into the wells of 96-well plates, grown to confluence and propagated for six splits. The plates were then assayed by the SSCA and wells containing PrP^res^-positive cells (spot numbers>[background+5 SDs]) were scored as positive. **B.** The ratio of validated prions/cells in the swa-containing plate to prions per cell in the swa-free plate yields the frequency of pre-existing swa-resistant prions in the population. Of those scored as positive in the presence of swa, seven AMO10-derived and seven PK1-derived prion populations were analyzed by the SSCA on PK1 cells in the absence or presence of swa to verify the true swa resistance of the prion population. In the case of RML^PK1^ propagated in the presence of swa, 0/7 positive wells, i.e. <14.3% contained swa-resistant prions; the corresponding value for RML^AMO10^ was 4/7, i.e. 57%. These values were used to recalculate the true frequencies in Table B.
(PDF)
