# Supplementary figures and images for: Acquisition of Drug Resistance and Dependence by Prions
Source: PLoS Pathog. 2013 Feb 7;9(2):e1003158. doi: 10.1371/journal.ppat.1003158 (PMC3567182; doi:10.1371/journal.ppat.1003158)

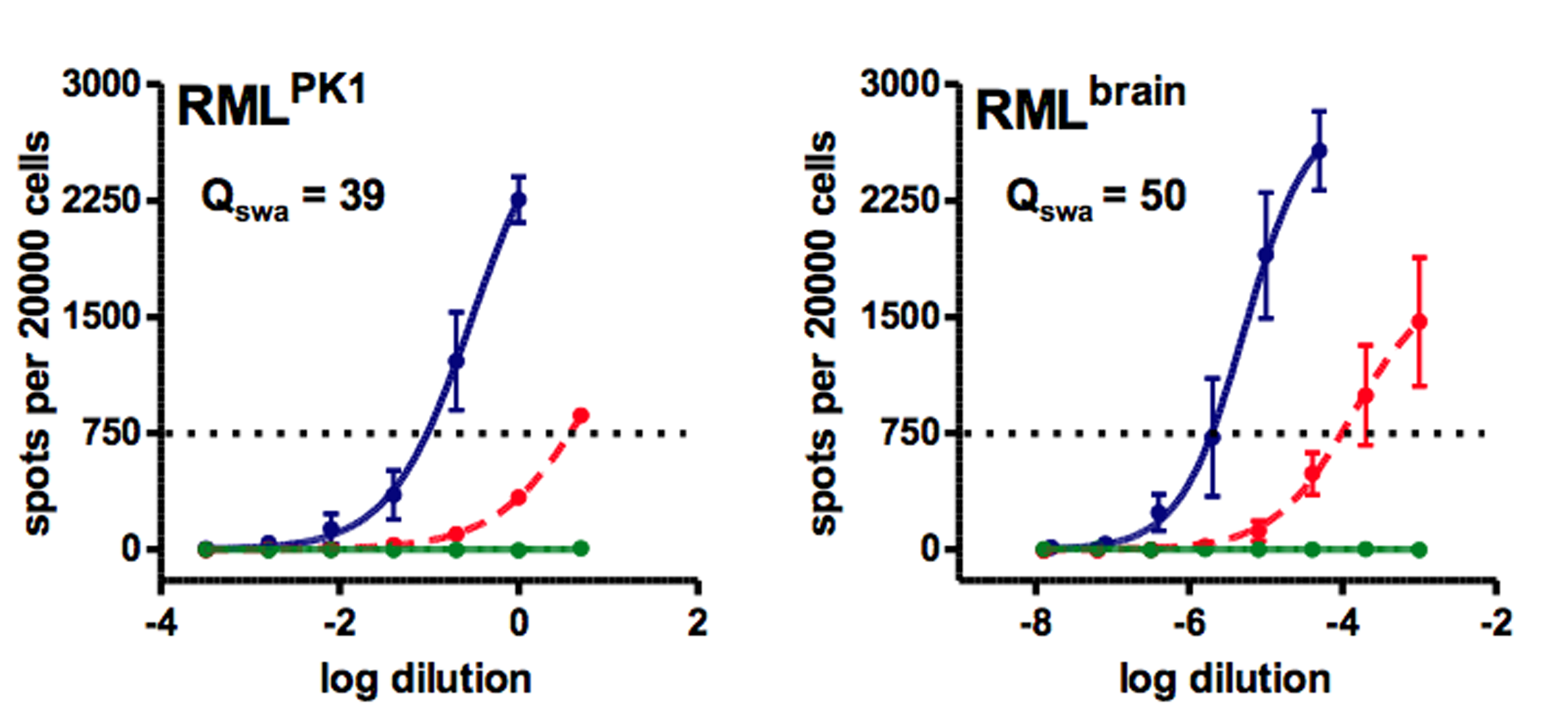

Supplement: Figure S1 — Brain and cell-derived RML prions are swa sensitive and R33 incompetent. When assayed by ECPA on R332H11 cells (green line) or on PK1 cells in the absence (blue line) or presence (red, dashed line) of swa, PK1 cell-derived and brain-derived RML prions show the same cell tropism pattern: Neither of the two prion populations is able to infect R332H11 cells and their propagation in PK1 cells is similarly inhibited by the presence of swa. RIs are the reciprocals of the dilutions required to yield 750 PrPres positive cells per 20000 cells. Qswa = RIPK1/RIPK1+swa indicates the inhibitory effect of swa on the analyzed prion sample. (TIF) [file ppat.1003158.s001.tif]

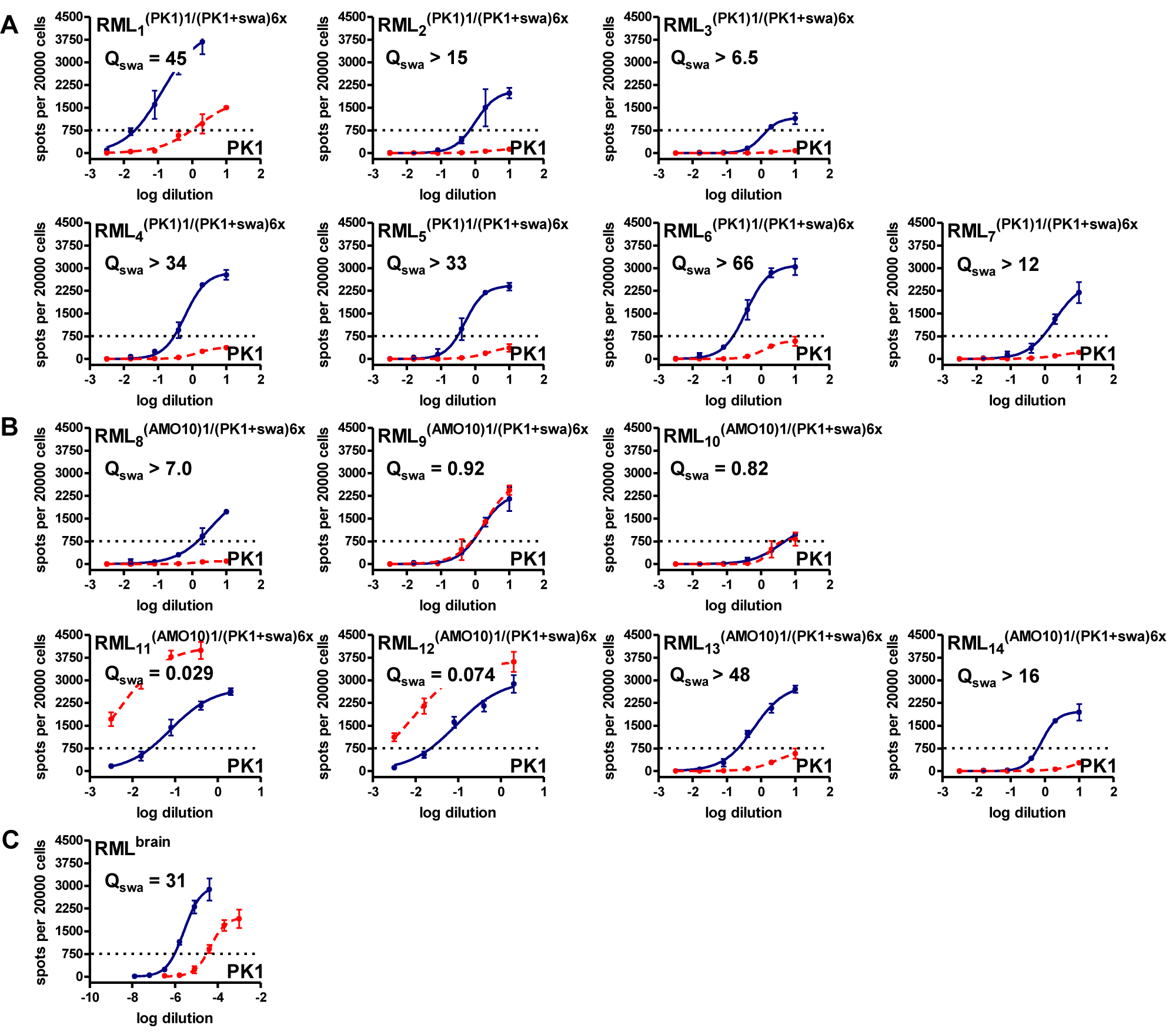

Supplement: Figure S2 — Validation of apparently swa-resistant prion populations recovered in the Frequency Assay on PK1- or AMO10-derived prions. Cells from seven positive wells obtained in the Frequency Assay (Table S1A) on PK1-derived and AMO10-derived populations were expanded, and concentrated CM was analyzed by the SSCA on PK1 cells in the absence (blue line) or presence (red, dashed line) of swa. RIs are the reciprocals of the dilutions required to yield 750 PrPres positive cells per 20000 cells. Qswa = RIPK1/RIPK1+swa reflects the inhibitory effect of swa on the analyzed prion sample and may be compared to the effect on brain-derived RML prions. A. All seven PK1-derived prion samples infected PK1 cells in the absence but not in the presence of swa. B. Three of the seven AMO10-derived prion populations were swa-sensitive prions (samples 8, 13, 14), two were swa resistant (samples 9, 10) and two were swa dependent (samples 11, 12). C. Brain derived RML prions were assayed as control. (TIF) [file ppat.1003158.s002.tif]

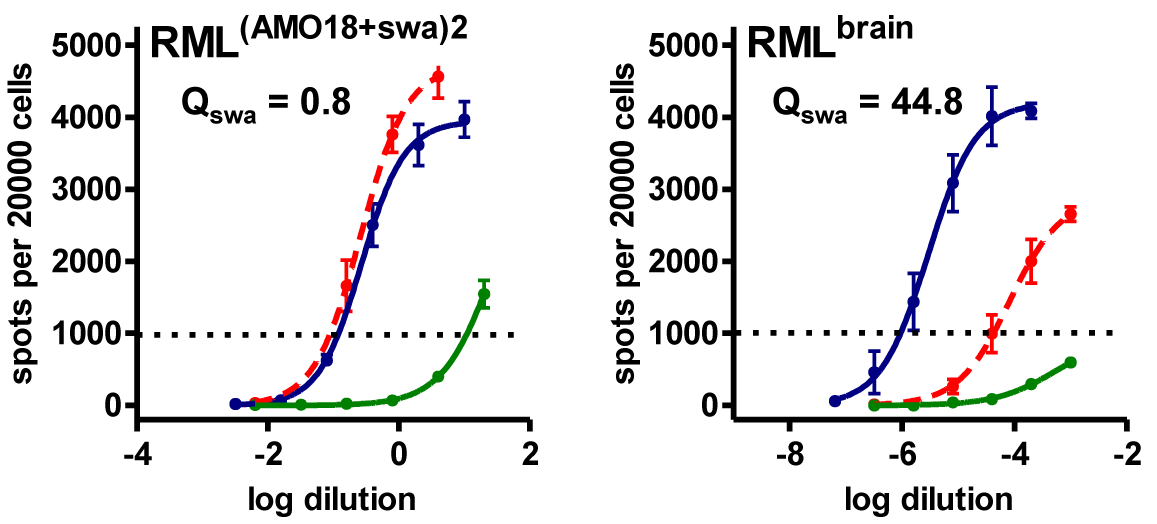

Supplement: Figure S3 — Selection of swa-resistant RML prions in PK18 cells. AMO18 cells were infected with RML prions, cultured in the presence of swa, and prions secreted into the conditioned medium (CM) were concentrated (CCM) and used to infect fresh batches of cells in the constant presence of swa. CCM recovered from this culture was analyzed by the SSCA on R332H11 cells (green line) as well as PK1 cells in the absence (blue line) or presence (red, dashed line) of swa (left graph). RIs are the reciprocals of the dilution yielding 1000 PrPres positive cells per 20000 cells. Qswa = RIPK1/RIPK1+swa indicates the inhibitory effect of swa on the analyzed prion sample and may be compared to the Qswa value of swa-sensitive brain-derived RML prions, assayed in parallel (right panel). Brain-derived RML prions are unable to infect R332H11 cells and their propagation in PK1 cells is strongly inhibited by swa. PK18-derived prions are inefficiently propagated by R332H11 cells but fully swa resistant. (TIF) [file ppat.1003158.s003.tif]

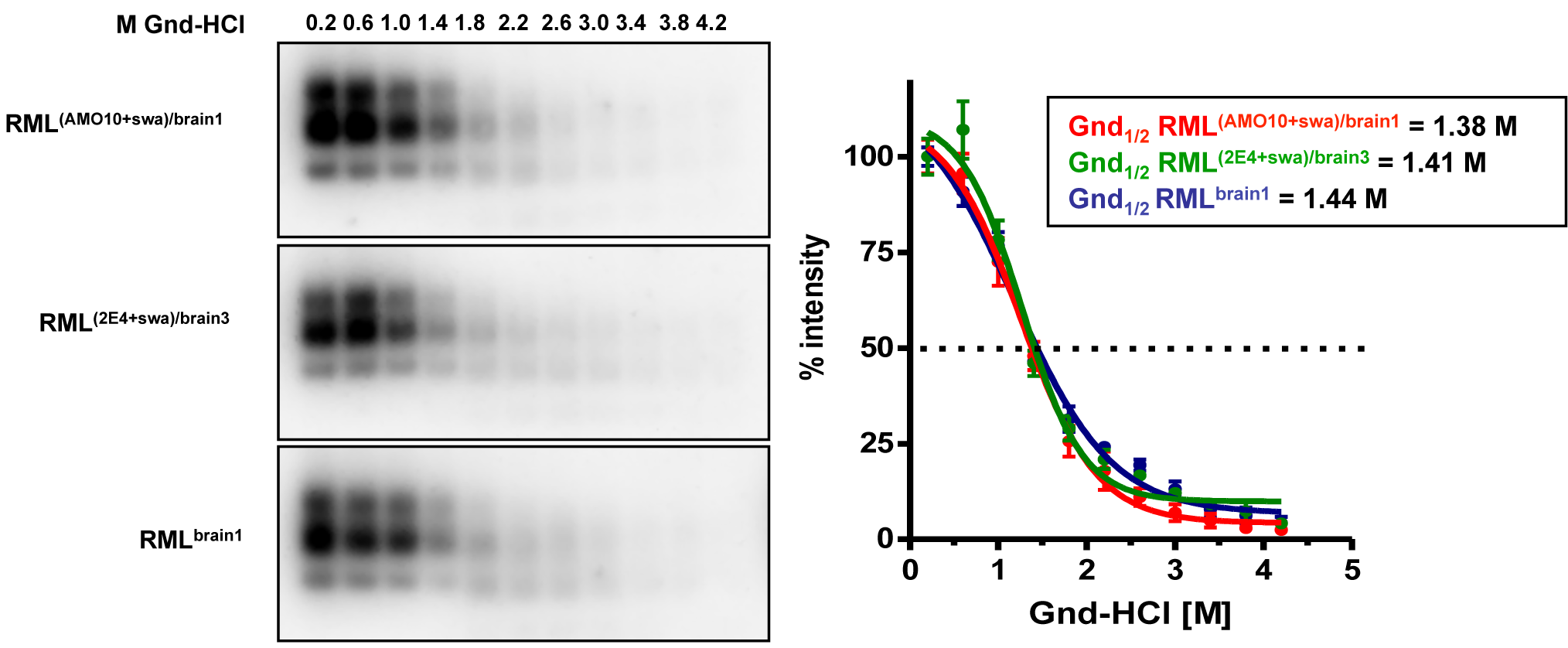

Supplement: Figure S4 — Conformational stability assay of brain homogenates. Swa-resistant AMO10-derived prions and swa-dependent 2E4-derived prions as well as brain-derived RML prions were propagated in mice. Brain homogenates of the three samples were adjusted to increasing concentrations of guanidine hydrochloride (Gdn.HCl) ranging from 0.2 M to 4.2 M, incubated for 15 minutes at 25°C, treated with proteinase K and precipitated with trichloroacetic acid. PrPres was detected by western blot analysis on triplicate gels, of which one representative blot is shown, and signals were expressed in percentage of the signal for 0.2 M Gnd.HCL. Gnd.HCl1/2, i.e. the molarity at which 50% of the PrPres became susceptible to PK digestion, was 1.4 M for all three preparations. (TIF) [file ppat.1003158.s004.tif]
