# Supplementary material for: Acquisition of Drug Resistance and Dependence by Prions
Source: PLoS Pathog. 2013 Feb 7;9(2):e1003158. doi: 10.1371/journal.ppat.1003158 (PMC3567182; doi:10.1371/journal.ppat.1003158)
Supplement: Table S1 — Quantification of preexisting swa-resistant prions in PK1- and AMO10-derived prion populations by the Frequency Assay. A. Conditioned medium recovered from PK1 and AMO10 cells, both inoculated with brain-derived RML prions in the absence of swa, was subjected to the Frequency Assay on PK1 cells: PK1 cells were infected in the presence or absence of swa with prions from one or the other source, and pools of 2000 cells (in the presence of swa) or of 10 cells (plus 1990 uninfected cells, in the absence of swa) were distributed into the wells of 96-well plates, grown to confluence and propagated for six splits. The plates were then assayed by the SSCA and wells containing PrPres-positive cells (spot numbers>[background+5 SDs]) were scored as positive. B. The ratio of validated prions/cells in the swa-containing plate to prions per cell in the swa-free plate yields the frequency of pre-existing swa-resistant prions in the population. Of those scored as positive in the presence of swa, seven AMO10-derived and seven PK1-derived prion populations were analyzed by the SSCA on PK1 cells in the absence or presence of swa to verify the true swa resistance of the prion population. In the case of RMLPK1 propagated in the presence of swa, 0/7 positive wells, i.e. <14.3% contained swa-resistant prions; the corresponding value for RMLAMO10 was 4/7, i.e. 57%. These values were used to recalculate the true frequencies in Table B. (PDF) [file ppat.1003158.s005.pdf]

A - Calculation prior to validation								
**swa**	**D**	**N**	**neg./total wells**	**$P_{m_w}(0)$**	**$m_w = \ln[1/ P_{m_w}(0)]$**	**$m_c = m_w/N$**	**m_c/D**	**Av ± SD**
**RML**[PK1]								
+	1	2000	121/440	0.275	1.3	0.00065	0.00065	0.00065
-	1	10	2/44	0.045	3.1	0.31	0.31	0.27 ± 0.13
-	0.2	10	31/44	0.7	0.35	0.035	0.18	
-	0.04	10	37/44	0.84	0.17	0.017	0.43	
-	0.008	10	87/88	0.99	0.011	0.0011	0.14	
-	0.0016	10	87/87	1	0	0	--------	
**$m_c/D_{[+swa]} / m_c/D_{[-swa]}$ = 0.0024**								
**RML**[AMO10]								
+	1	2000	387/434	0.89	0.12	0.000057	0.000057	0.000057
-	1	10	31/43	0.72	0.33	0.033	0.033	0.041 ± 0.11
-	0.2	10	39/43	0.91	0.1	0.01	0.049	
-	0.04	10	43/43	1	0	0	--------	
-	0.008	10	87/87	1	0	0	--------	
-	0.0016	10	87/87	1	0	0	--------	
**$m_c/D_{[+swa]} / m_c/D_{[-swa]}$ = 0.0014**								

B -Calculation after validation								
**swa**	**D**	**N**	**neg./total wells**	**$P_{m_w}(0)$**	**$m_w= \ln[1/ P_{m_w}(0)]$**	**$m_c= m_w/N$**	**m_c/D**	**Av ± SD**
**RML**[PK1]								
+	1	2000	>394/440	>0.90	<0.11	<0.000055	<0.000055	<0.000055
-	1	10	Feb-40	0.045	3.09	0.31	0.31	0.27 ± 0.13
-	0.2	10	31/40	0.7	0.35	0.035	0.18	
-	0.04	10	36/44	0.84	0.17	0.017	0.43	
-	0.008	10	87/88	0.99	0.01	0.0011	0.14	
-	0.0016	10	87/87	1	0	0	0	
**$m_c/D_{[+swa]} / m_c/D_{[-swa]}$ < 0.00020**								
**RML**[AMO10]								
+	1	2000	407/434	0.94	0.06	0.000032	0.000032	0.000032
-	1	10	31/43	0.72	0.33	0.033	0.033	0.041 ± 0.011
-	0.2	10	39/43	0.91	0.1	0.0098	0.049	
-	0.04	10	43/43	1	0	0	0	
-	0.008	10	87/87	1	0	0	0	
-	0.0016	10	87/87	1	0	0	0	
**$m_c/D_{[+swa]} / m_c/D_{[-swa]}$ = 0.00078**								

D: dilution; **N:** cells/well; **$Pm_w(0)$:** probability of a well remaining uninfected = e^{-m} = 1-(positive wells/total wells); **m_w:** calculated prions/well; **m_c:** calculated prions/cell; **$m_{c/D}$:** calculated prions per cell corrected for dilution; **$m_{c/D[+swa]}/m_{c/D[-swa]}$:** frequency of swa- resistant prions in population
